# Commentary: Perceived social support and health-related quality of life in AYA cancer survivors and controls

**DOI:** 10.3389/fpsyg.2017.00225

**Published:** 2017-02-16

**Authors:** Peter Krajmer, Milica Malá, Cindy Bahy, Radoslav Blaho, L'ubomír Harinek, Júlia Horáková, Alexandra Kolenová

**Affiliations:** ^1^Pediatric Haematology and Oncology Clinic, Children's University HospitalBratislava, Slovakia; ^2^Department of Psychology, Faculty of Arts, Comenius UniversityBratislava, Slovakia; ^3^Department of Special Education, Faculty of Education, Comenius UniversityBratislava, Slovakia; ^4^Faculty of Medicine, Comenius UniversityBratislava, Slovakia

**Keywords:** AYA cancer survivors, social support, HRQoL, mixed research methods, psychotraumatic experience

In a recent study Tremolada et al. (2016) surveyed perceived social support of adolescents and young adults' (AYA) cancer survivors as compared to a control group in North Eastern Italy. Their findings include cancer survivors having better quality of life despite a lower level of perceived social support provided by family, friends, and significant others compared with an AYA peer control group. Research identified those peculiarities using well-established quantitative methods. However, this study did not provide direct opinions from AYA survivors on the subjective criterias they considered while evaluating the social support they received. In this commentary we examine using qualitative narratives as a beneficial complementary method to investigate the reasons attributed by AYA survivors to their perceived lack of social support and get a better understanding of this phenomenon.

Quantitative research methods have been widely used in the evaluation of quality of life of cancer patients. In their research Tremolada et al. (2016) showed that AYA survivors reported “less support, particularly from family and significant others, even if they declared higher level of functioning on the Social Scale of SF-36.” They also had a higher HRQoL. She used 2 self-reported questionnaires: Short-Form Health Survey (SF-36) and Multidimensional Scale of Perceived Social Support for measuring social support. The evaluation of health quality of life and perceived social support being sensitive to sociocultural factors. We will likely see different trends in various countries. Research conducted in Czech and Slovak Republic observed that AYA survivors had higher HRQoL compared to controls (Žilínek et al., 2013; Koutná et al., 2014). Ex-patients had also higher level of perceived social support (Žilínek et al., 2013; Koutná et al., 2014). For methodological reasons, those results, are to be considered with reserves (small sample size, including criteria differences and use of different questionnaires). Those researches did not reveal the reasons attributed by survivors in their appreciation of life and social support. As Tremolada et al. (2016) noted: “Using qualitative methodology such as semi-structured interviews would be able to give direct voice to AYAs.”

Emergence of medical patient-centered models of care has encouraged the development of “mixed methods” of research in the field of human sciences. Those “mixed methods” synerge the utilization of data collected with quantitative and qualitative methods (Creswell et al., 2004). They are used to promote a reflection on participants' point of view, foster interaction between researchers, and collect comprehensive data (Wisdom and Creswell, 2013). A research conducted in Slovakia applied mixed methods to examine the quality of life of AYA cancer survivors. They used qualitative data to explore the meanings survivors attributed to their relative higher scores on HRQoL scales (Blaho et al., 2015). This study noted that hemato-oncological diagnoses were very traumatic events in the lives of AYA survivors. Even 5 years after treatment they still perceived their lives as splitted into two realities before and after the illness. Whether they were doing well socially or not they explained it as being “despite” or “due” to their experience with a serious illness. Therefore, AYA survivors' current situation could either be understood as Post-traumatic growth or PTSD.

AYA cancer survivors who had lower scores of perceived social support, often reported being discriminated and socially isolated because they have had a serious illness. They felt misunderstood by their community. In AYA' search for meaning they mentioned feeling blamed and accused of using their illness for perks, people being jealous of them for getting more attention from adults or having missed school. They thought they may be rejected due to their changed physical appearance or different center of interests: “*My classmates didn't want to understand why I changed so much, my body, my appearance” (female 13)*. Finally they were uncomfortable with feeling that others had pity for them. The information collected with this added qualitative study were helpful to clinical psychologists working in pediatric hemato-oncology departments in Bratislava, Slovakia. They were able to discuss targeted interventions to offer higher quality support to patients and their families during and after treatment. Adding qualitative data to quantitative research was beneficial to patients. It is our belief that research in the field of psychology can guide clinical psychologists in their work and that feedback from those clinicians could determine further research. Using mixed methods of investigation seems to be a good approach to facilitate this alliance.

Psychosocial well-being of pediatric patients during and after oncologic treatment is of multidimensional complexities. Cancer and its treatment affect not only their physical but also their emotional, cognitive, social, and spiritual health. Minimizing the traumatic impact of such illnesses and treatments is part of a patient-centered approach to medical care. In their article Tremolada et al. (2016) used quantitative methods and showed that AYA survivors had a higher HRQoL compared to control. At the end of their report they mentioned the benefits of using qualitative narratives to question and advance their findings. In this commentary we presented a research conducted in Slovakia (Blaho et al., 2015). They used mixed methods to study HRQoL including perceived social support. Those authors showed that higher HRQoL was inseparable from AYA cancer survivors' traumatic experience with a serious illness. Survivors who had a lower perceived level of social support understood it as a consequence of oncological treatment and its late effects. Thanks to those results psychologists investigated potential interventions to improve the quality of life of pediatric oncological patients during and after treatment. Using mixed methods of research can therefore be beneficial not only to the advancement of science but also to clinical practice.

## Author contributions

PK designed and supervised the project. Wrote part of the commentary. Revised the manuscript. MM, CB, RB, and LH participated equally in writing and editing the manuscript. JH and AK: Made a critical revision of the article and gave final approval of the version to be published.

### Conflict of interest statement

The authors declare that the research was conducted in the absence of any commercial or financial relationships that could be construed as a potential conflict of interest.
